# Comparative Study of Interactions between Human cGAS and Inhibitors: Insights from Molecular Dynamics and MM/PBSA Studies

**DOI:** 10.3390/ijms22031164

**Published:** 2021-01-25

**Authors:** Xiaowen Wang, Wenjin Li

**Affiliations:** 1 Institute for Advanced Study, Shenzhen University, Shenzhen 518060, China; wxw@szu.edu.cn; 2 College of Physics and Optoelectronic Engineering, Shenzhen University, Shenzhen 518060, China

**Keywords:** cGAS, inhibitor, MM/PBSA, MD simulation, sandwiched structures

## Abstract

Recent studies have identified cyclic GMP-AMP synthase (cGAS) as an important target for treating autoimmune diseases, and several inhibitors of human cGAS (hcGAS) and their structures in complexation with hcGAS have been reported. However, the mechanisms via which these inhibitors interact with hcGAS are not completely understood. Here, we aimed to assess the performance of molecular mechanics/Poisson–Boltzmann solvent-accessible surface area (MM/PBSA) in evaluating the binding affinity of various hcGAS inhibitors and to elucidate their detailed interactions with hcGAS from an energetic viewpoint. Using molecular dynamics (MD) simulation and MM/PBSA approaches, the estimated free energies were in good agreement with the experimental ones, with a Pearson’s correlation coefficient and Spearman’s rank coefficient of 0.67 and 0.46, respectively. In per-residue energy decomposition analysis, four residues, K362, R376, Y436, and K439 in hcGAS were found to contribute significantly to the binding with inhibitors via hydrogen bonding, salt bridges, and various π interactions, such as π· · ·π stacking, cation· · ·π, hydroxyl· · ·π, and alkyl· · ·π interactions. In addition, we discussed other key interactions between specific residues and ligands, in particular, between H363 and JUJ, F379 and 9BY, and H437 and 8ZM. The sandwiched structures of the inhibitor bound to the guanidinium group of R376 and the phenyl ring of Y436 were also consistent with the experimental data. The results indicated that MM/PBSA in combination with other virtual screening methods, could be a reliable approach to discover new hcGAS inhibitors and thus is valuable for potential treatments of cGAS-dependent inflammatory diseases.

## 1. Introduction

Free DNA in the cytosol is detected by a type of nucleotidyltransferase called cyclic GMP-AMP (cGAMP) synthase (cGAS) [1]. Upon binding to DNA, cGAS is activated and produces cGAMP from GTP and ATP. The second messenger, cGAMP, activates and binds the stimulator of interferon genes, which subsequently induces the secretion of type I interferons and triggers the downstream innate immune response [2,3,4,5]. While recognition of pathogen DNA is essential for host defense against infections, aberrant activation of cGAS may trigger autoimmune diseases such as systemic lupus erythematosus and Aicardi–Goutières syndrome in the presence of self-DNA [6,7], which may be displaced nuclear or mitochondrial DNA generated as by-products of cellular damage. Hence, cGAS is a vital drug target for treating autoimmune diseases and for preventing autoinflammation in therapeutic strategies against cancers.

Experimental studies have shown that knockout of the cGAS gene in *Trex1^−/−^* mice dramatically reduces tissue inflammation and hinders autoantibody production [8,9]. Thus, inhibition of cGAS activity is a promising therapeutic strategy for autoimmune diseases. Recently, several inhibitors of human (h) and mouse (m) cGAS have been reported [10,11,12,13]. All of them bind to the catalytic center of cGAS and suppress its nucleotidyltransferase activity. RU.521 is a promising inhibitor (IC_50_ = 0.11 µM) of mcGAS that can regulate the levels of interferon; however, it binds to hcGAS with low affinity (IC_50_ = 2.94 µM) in spite of approximately 60% sequence identity between hcGAS and mcGAS [14]. A high-throughput screening study showed that the compounds G108 (containing a pyrazole group) and G105 (containing a 2-amino pyridine ring) were novel specific inhibitors of hcGAS, which targeted the identical activation loop region containing R376 and Y436, similar to the binding pathways of 2,3-cGAMP [12]. While G150 and G108 showed high binding affinities in complexes with hcGAS, with IC_50_ values of 10.2 nM and 27.5 nM, respectively, they were both inactivated during assessment with mcGAS [12]. Another high-affinity inhibitor, PF–06928215, which binds to the hcGAS active sites, was shown to have a dissociation constant (*K*_d_) of 200 nM (IC_50_ = 4.9 µM) using high-throughput screening assays [11]. Based on the inactive PF–06928215 in cell-based cGAS, three potent compounds, 18, S2, and S3 (IC_50_ = 29.88 ± 3.20, 13.1 ± 0.09, and 4.9 ± 0.26 µM), with highly consistent binding modes, were identified by Zhao and co-workers [13]. More information regarding various hcGAS inhibitors as therapeutic targets of interest are presented in Table S1.

To complement and validate the results obtained using an experimental approach, computational modeling, molecular dynamics (MD) simulations, and molecular mechanics/Poisson–Boltzmann solvent-accessible surface area (MM/PBSA) can be used for analyzing the real-time dynamic behavior of biomolecules and predicting the binding free energy. These methods have been widely used for elucidating the binding mechanism and interaction modes of protein-inhibitor systems [15,16,17]. Recently, virtual screening was performed for the first time, combined with MD and modeling methods, to identify new hcGAS-binding inhibitors [13]. However, complete understanding regarding the molecular mechanism of hcGAS-inhibitor interactions, especially from the viewpoint of the dynamics and energy of the interactions, is still lacking.

**Figure 1:**
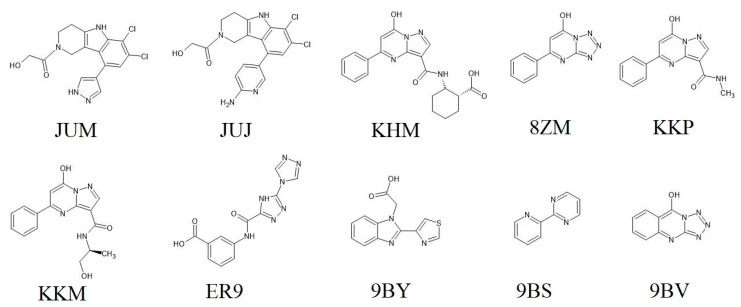
Chemical structures of the hcGAS-binding inhibitors used in this study with their protein data bank (PDB) names. Tricyclic pyridoindole core for JUM (pyrazole ring) and JUJ (2-amino pyridine ring), diazolo-pyrimidine aromatic core for KHM, KKP, and KKM, tetrazolo-pyrimidine aromatic core for 8ZM and 9BV (thiazole ring), bi (1, 2, 4-triazole) core for ER9, tricyclic benzimidazole core for 9BY, and pyridine and pyrimidine rings for 9BS are shown.

In this study, we aimed to assess the performance of MM/PBSA in evaluating the binding affinity of 10 hcGAS inhibitors (listed in Figure 1 and Table S1). In addition, we aimed to elucidate the binding modes of hcGAS with these inhibitors in combining MD simulations and per-residue energy decomposition analysis and compare these observations with those obtained using experimental methods. In combination with existing virtual screening approaches, our study could provide theoretical guidance in the search for new cGAS inhibitors and thus be helpful in the treatment of autoimmune diseases.

## 2. Results and Discussion

### 2.1. Structural Analysis

Before performing the MM/PBSA analysis, it was necessary to assess the convergence of the sampled structures during MD simulations [18]. The root mean square deviation (RMSD) of the 10 hcGAS-inhibitor complexes as a function of time are plotted in Figure S1. Simultaneously, Table 1 lists the average RMSD values for isolated hcGAS and inhibitors, including the zinc-thumb domain and whole systems. As shown in Figure S1, the RMSDs of all the hcGAS proteins and inhibitors fluctuated within 0.3 and 0.15 nm, respectively. In particular, 9BS-bound hcGAS showed higher fluctuation with an average RMSD of 0.274 ± 0.030 nm than 9BY and 9BV-bound hcGAS (Figure S1a). The inhibitors 9BS and 9BV displayed more accurate structural overlap with the experimental ones compared to other inhibitors, with RMSD values of 0.017 ± 0.046 and 0.014 ± 0.049 nm, respectively; 9BY showed larger RMSD distribution of 0.089 ± 0.029 (Figure S1b). JUM and ER9 show relatively larger RMSD fluctuations in the range of 0.1−0.15 nm within 7 ns than the other inhibitors (Figure S1b), while reaching final equilibrium with the average RMSD values of 0.097 ± 0.044 and 0.116 ± 0.056 nm, respectively (Table 1). The zinc-thumb domain plays a key role in maintaining cGAS recognition, which was evident from the RMSD values lesser than 0.05 nm for all systems (see Table 1). Overall, these plots suggested that the inhibitors stayed in the preferred positions in the binding pockets and that the entire hcGAS-inhibitor complex remained well balanced during the MD simulations. To further confirm that the protein-ligand complexes were converged within the 20 ns MD simulations, one of the three parallel simulations for each inhibitor was extended to 50 ns, and comparable RMSDs were observed as to the ones within the previous 20 ns simulations (Figure S2).

### 2.2. Binding Free Energy

MM/PBSA analysis of three parallel trajectories was used for determining the free energies of hcGAS binding with various inhibitors (see Table 2). The binding free energies were also estimated from each simulation separately (Tables S2–S4), and they were strongly correlated with the one averaged from all three simulations (see Figure S3), demonstrating the consistency of the results from independent simulations. The reported experimental data on IC_50_ and *K*_d_ of the inhibitors were used to evaluate the performance of MM/PBSA calculations. For conveniently comparing the binding affinities between the complexes, we classified the hcGAS-binding inhibitors into three categories (see Table S1): Tricyclic pyridoindole core of JUM and JUJ (I) [12], diazolo-/tetrazolo-pyrimidine aromatic core of KHM, 8ZM, KKP, and KKM (II) [11], and the other three inhibitors, including various heterocycles of 9BY, 9BS, and 9BV (III) [10]. It is noteworthy that inhibitor ER9 contains a bi(1,2,4-triazole) core, which is a novel inhibitor obtained from virtual screening based on the inhibitor KHM [13], and was hence placed in category II for analysis.

**Figure 2:**
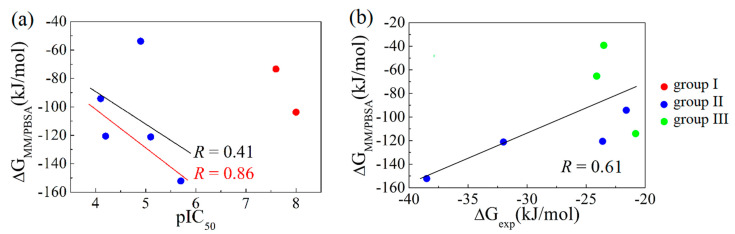
Correlations between available pIC_50_/experimental binding free energies and the averaged binding free energies estimated using the MM/PBSA method for ligand (**a**) in groups I and II, and (**b**) in groups II and III. The three ligand categories are distinguished by red dots for group I, blue dots for group II, and green dots for group III, respectively. In (**a**), the black line fits to all the data points of group II, while the red line to the ones for the group II but ER9. In (**b**), the black line fits to all the data points.

As shown in Table 2, the binding free energies predicted using MM/PBSA are in accordance with the experimentally determined affinities. In terms of the IC_50_ values, the binding affinity of JUJ-bound hcGAS was stronger than that of JUM-bound hcGAS (0.0102 and 0.0275 μM, respectively). This was well predicted from the calculated binding free energies of −103.8 ± 5.5 kJ/mol and −73.4 ± 5.3 kJ/mol. MM/PBSA also predicted the order of the binding free energy of the inhibitors with diazolo-/tetrazolo-pyrimidine aromatic core to be KHM > KKM > KKP > 8ZM, which is consistent with the IC_50_ values of 2.0, 8.1, 69.0, and 78.0 μM, respectively. Although the IC_50_ (13.1 μM) of ER9 in the enzyme activity assay was stronger than that of KKP (69 μM) determined using a fluorescence polarization assay [11,13], it is difficult to compare their binding affinities owing to the use of different approaches. In addition, the calculated binding free energies of 9BS and 9BV bound to hcGAS were −65.3 ± 3.6 and −39.2 ± 3.6 kJ/mol, respectively, with dissociation constant *K*_d_ of 64 ± 3 and 80 ± 4, respectively. The experimental data suggested that the affinity of 9BY was weaker than those of 9BS and 9BV, although the calculated binding free energy of the hcGAS–9BY interaction (−114.1 ± 4.3 kJ/mol) was predicted to be more than that of 9BS and 9BV. We further evaluated the correlation of the binding free energy between the pIC_50_/∆*G*_exp_ and MM/PBSA calculation. It should note that the IC_50_ or *K*_d_ values for these three groups of ligands were obtained from different experimental methods, more specifically, high-throughput screening assay for ligand group I [12], fluorescence polarization assay for ligand group II excluding ER9 [11], enzyme activity assay for ER9 [13], and a novel SPR-based enzymatic assay for ligand group III [10]. Since the value of IC_50_ was dependent on the enzyme concentration used in the experiments, the correlation between pIC_50_ and the predicted ∆*G*_MM/PBSA_ was thus discussed separately for inhibitors in different groups. As shown in Figure 2a, the predicted binding free energy for groups I and II negatively correlated with the experimental values, especially for the ligand in group II excluding ER9 a good correlation coefficient of 0.86 was obtained. As shown in Figure 2b, the correlation between ∆*G*_exp_ and ∆*G*_MM/PBSA_ for groups II and III was as high as 0.61. Note that the RMSD of both ER9 and 9BY (Figure S1) were significantly larger than the ones of other inhibitors, which might affect the reliability of their predicted ∆*G*_MM/PBSA_ and resulted in an underestimated correlation. Thus, the above results suggested that MM/PBSA could be a promising and cheap approach to estimate the binding affinity of potential inhibitors of hcGAS.

The averaged binding free energies at different time scales from three parallel trajectories for the 10 hcGAS-inhibitor systems obtained using MM/PBSA analysis are shown in Table 3. Compared to the experimental binding affinities, most calculated binding free energies (∆*G*_bind_) are generally overestimated, similar to the results of other studies on protein-ligand interactions [19,20]. The Spearman’s and Pearson’s correlation coefficients are also provided here to evaluate the ranking of the binding free energies and their correlation with experimental data [21,22]. As listed in Table 3, the Pearson’s correlation coefficient (*r*) was 0.52 at the early simulation stage (0−4 ns), with Spearman’s rank correlation coefficient (*ρ*) of 0.32, while the studies on dynamics predicted relatively stronger correlations and fluctuation in the vicinity of 0.66 for *r*. Furthermore, the rankings of the binding free energies remained constant at *ρ* = 0.46 after 8 ns. In addition, we inspected the various energy distributions as a function of time, as shown in Figure S4. The components ∆*E*_vdW_ and ∆*G*_np/solv_ were generally stabilized during the entire trajectories. However, the electrostatic and polar solvation energies showed larger fluctuations in hcGAS binding with JUJ, KHM, and KKM than the other inhibitors, leading to binding free energies with stronger disturbance. Despite the limitations of MM/PBSA studies, which were mainly due to force field accuracy, polar contribution to solvation, entropy estimation, and sampling conditions, this method was used as a potential tool for predicting the relative binding free energy of protein-ligand interactions [23,24].

### 2.3. Energy Decomposition Analysis

Energy decompositions for 10 hcGAS-inhibitor interactions are shown in Table 2. vdW (∆*E*_vdW_), electrostatic energy (∆*E*_ele_), and nonpolar solvation energy (∆*G*_np/solv_) contributed to attractive interactions, while polar solvation energy (∆*G*_pb/solv_) contributed repulsively to the total binding free energies. Similar to the findings of Zhao and co-workers [13], the total nonpolar energies (∆*G*_np_) were stronger than the total polar energies (∆*G*_pb_), indicating that total nonpolar components in the hcGAS-inhibitor interactions contributed more significantly than total polar interactions. In terms of attractive contributions, vdW and electrostatic interactions contributed the most to binding free energies, especially for the inhibitors KHM, 9BY, KKM, and ER9. For the hcGAS-9BV and hcGAS-8ZM interactions, the vdW contributions exceeded −110 kJ/mol, while the electrostatic interactions provided moderate contributions under −30 kJ/mol, comparable to the nonpolar solvation energies. The nonpolar energy of 9BY (∆*G*_np_ = −145.3 ± 1.5 kJ/mol) established stronger affinities than those of 9BS and 9BV mentioned above.

### 2.4. Single Residue Energy Analysis

**Figure 3:**
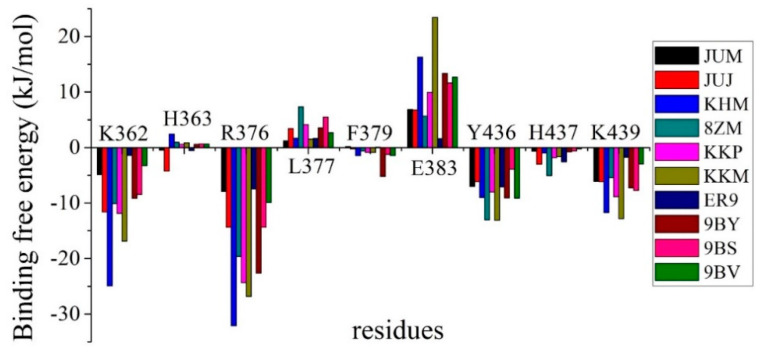
Binding free energies of the residues with major contributions within 6 Å of inhibitors in all hcGAS-inhibitor interactions.

The binding free energy of single amino acids in each system was determined to identify the residues that contributed considerably to the binding and the similarities and differences in the binding of the 10 inhibitors to the hcGAS protein. We evaluated the residues distributed within 0.6 nm from any atom in the inhibitor, the per-residue binding free energies of which are listed in Table S5. Among these amino acids, we selected and focused on the residues that contributed significantly to the binding free energy (Figure 3). K362, R376, Y436, and K439 contributed attractively to the hcGAS-inhibitor interactions, except for the binding of K362 and K439 with ER9. In addition, E383 participated in more repulsive interactions with most inhibitors than the other residues, with a small binding free energy of 1.60 kcal/mol with ER9 (see Table S5). L377 in hcGAS also contributed repulsively to its binding with various inhibitors, especially in the JUJ, 8ZM, KKP, 9BY, 9BS, and 9BV-binding systems. Compared to others, only the inhibitors JUJ, 9BY, and 8ZM contributed distinctly to binding with H363, F379, and H437.

### 2.5. Binding Modes of the Inhibitors with hcGAS

Figure 3 clearly indicates that the polar amino acids K362, R376, and K439, and the aromatic amino acid Y436, interacted primarily with the inhibitors, which were in good agreement with the experimental results [10,11,12,13]. Hence, we focused on the binding modes of these four key residues with various inhibitors in the active site based on MM/PBSA and MD analyses.

#### 2.5.1. Role of K362

**Figure 4:**
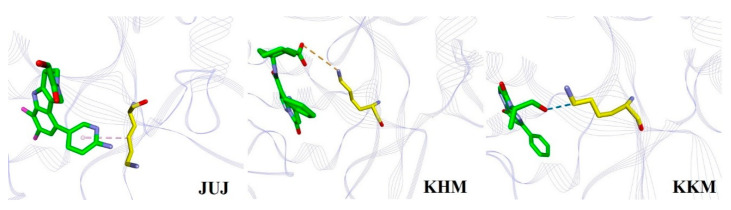
Binding of three inhibitors, JUJ, KHM, and KKM, with K362. The C atoms of inhibitors and residues are shown in green and yellow, respectively. The N, O, and Cl atoms are shown as blue, red, and magenta, respectively. All the hydrogen atoms are hidden for clarity. The proteins are displayed as purple line ribbon. The light pink, orange, and blue dashes represent the C−H· · ·π, salt bridge, and H-bonded interactions, respectively.

The predicted binding free energies of K362 with the inhibitors JUJ, KHM, 8ZM, KKP, KKM, 9BY, and 9BS were significantly more attractive than –8.0 kJ/mol (see Figure 3 and Table S5). Among them, maximum interaction was observed between K362 and KHM, with a binding free energy of −24.90 kJ/mol. Figure 4 shows that this strong attractive energy was mainly due to the salt bridge interaction formed by the carboxyl group of KHM and the side chain of K362, which was in agreement with the experimental observation regarding the vital interaction between the carboxylate headpiece of KHM and the polar residue K362, the latter playing a key role in maintaining the cGAS linear intermediate [11]. Second in ranking was the binding free energy of KKM (−16.84 kJ/mol), where the O−H group of KKM was suggested to form one H-bonded contact with the C−H group of the side chain of K362 (see Figure 4), although this interaction was not reported in a recent structural study [11]. Here, another novel C−H · · · π interaction was predicted between the side chain of K362 and the pyridine ring of JUJ, with a binding free energy of −11.61 kJ/mol. Although K362 in the hcGAS-KKP complex was predicted to have a favorable binding energy of −11.84 kJ/mol, no contact between K362 and KKP was formed in either the simulation structure or the crystal one [11], which was similar to the interactions between K362 and additional inhibitors JUM, 8ZM, ER9, 9BY, 9BS, and 9BV (see Figure S5). Among these inhibitors, only 8ZM, 9BY, 9BS, and 9BV were in loose contact with the polar group head of K362.

#### 2.5.2. Role of R376

**Figure 5:**
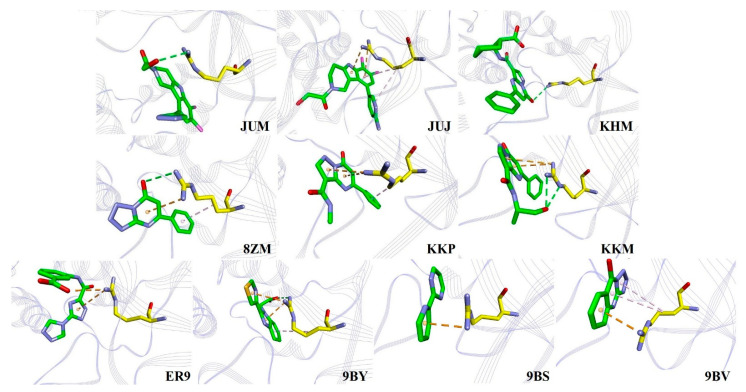
The modes of binding of the 10 inhibitors with R376. The C atoms of the inhibitors and R376 are shown in green and yellow, respectively. The N, O, S, and Cl atoms are shown in blue, red, orange, and magenta, respectively. All the hydrogen atoms are hidden for clarity. The proteins are displayed as purple line ribbon. The green, orange, and light pink dashes represent the H-bonded, π· · ·cation or salt bridge, and C−H· · ·π interactions, respectively.

Figure 5 clearly shows that hcGAS R376 plays a vital role in binding to any inhibitor and tends to use its guanidinium group with the five- or six-membered rings of heterocyclic inhibitors, forming important cation· · ·π interactions, and affecting molecular recognition, biological structures, and functions [25,26]. For example, two cation· · ·π interactions were formed by the N atom of the guanidinium group in R376 in complex with the diazolo-pyrimidine aromatic core of KKP and KKM (–24.33 and –26.80 kJ/mol), while two N atoms of the guanidinium group in R376 bound with the five- and six-membered rings of heterocyclic 9BY and JUJ (–22.63 and –14.38 kJ/mol), respectively. Figure 5 also shows that R376 uses an N atom of the guanidinium group to form one cation· · ·π interaction with the 1,2,4-triazole core of ER9 (–7.45 kJ/mol) and six-membered rings of 8ZM (–19.60 kJ/mol), 9BS (–14.36 kJ/mol), and 9BV (–9.90 kJ/mol), respectively. These structural interactions are consistent with the experimental observations [10,12,13]. In addition, hydrogen bonding interactions were observed in KHM (–32.09 kJ/mol), JUM (–7.88 kJ/mol), 8ZM, KKM, and 9BY (–22.63 kJ/mol) in complex with the side chain of R376. Salt bridge interaction was only observed between the guanidinium group of R376 and the carboxyl group of the ER9 interaction. Simultaneously, C−H · · · π interactions were also predicted to be involved in the binding of R376 with inhibitors such as JUJ, 8ZM, KKP, 9BY, and 9BV.

#### 2.5.3. Role of Y436

**Figure 6:**
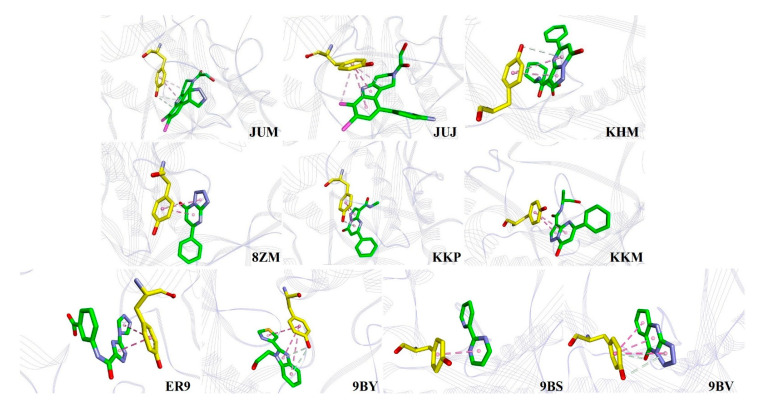
Binding of the 10 inhibitors used in this study with Y436. The C atoms of inhibitors and Y436 are shown in green and yellow, respectively. The pink and white dashes represent the π· · ·π stacking and O−H· · ·π interactions, respectively. The light pink dashes in JUM and JUJ show the C−H· · ·π interactions. The schemes of the atom and protein colors are the same as in Figure 5.

The hcGAS-bound inhibitors contain multiple aromatic rings (see Figure 1), which led to direct π· · ·π stacking interactions with Y436 at the active site, as shown in Figure 6. Simultaneously, O−H · · · π interactions were often observed when Y436 contacted the inhibitors KHM, JUM, 8ZM, KKP, 9BY, and 9BV. For instance, Y436 interacted with three aromatic rings of 9BV and 9BY in the form of π · · · π stacking interactions, consistent with experimental observations [10]. Although two O−H · · · π interactions were present in the 9BY-Y436 interaction, the free energies of binding of the two inhibitors, 9BV and 9BY, with Y436 were −9.09 and −9.07 kJ/mol (see Table S5), respectively. Second, Y436 formed two π· · ·π stacking interactions with the five- and six-membered heterocyclic rings of the inhibitors KHM (–8.98 kJ/mol), JUJ (–6.15 kJ/mol), KKM (–13.09 kJ/mol), 8ZM (–13.03 kJ/mol), and KKP (–8.00 kJ/mol), where a small hydrophobic pocket was observed between KHM and Y436 [11]. Among these, the structures of 8ZM, KKM, and KKP were similar, while the free energy of KKP binding to Y436 was predicted to be −5.0 kJ/mol weaker than that of 8ZM and KKM binding to Y436 (see Table S5). In addition, Y436 formed π · · · π interactions with two five-membered rings of ER9 (–7.08 kJ/mol), which was in good agreement with the experimental data [13]. Furthermore, only one π· · ·π stacking was found in the Y436-JUM/9BS interactions. Two additional O−H · · · π and one C−H · · · π interactions in the JUM-Y436 contact, with free energy of –7.00 kJ/mol, were also observed.

#### 2.5.4. Role of K439

The binding modes of K439 with the 10 inhibitors are shown in Figure S6. No evident contacts, such as hydrogen bonding or π−interactions, were observed in K439 binding with the inhibitors, which was consistent with experimental observations [10,11,12,13]. However, comparable free energies of binding with K439 were observed for KHM and KKM (−12.0 kJ/mol), JUM, JUJ, 8ZM, KKP, 9BY, and 9BS (−5.42 to −8.86 kJ/mol), and ER9 and 9BV (−2.0 kJ/mol) (Table S5), indicating the importance of K439 in interactions with the inhibitors, especially KHM and KKM, during binding.

**Figure 7:**
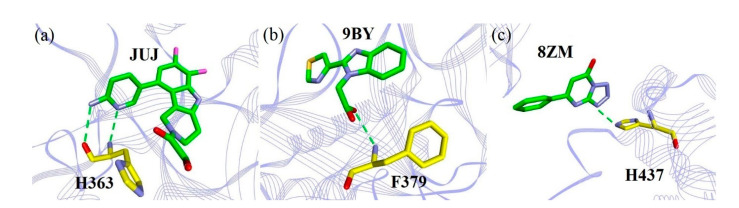
The binding details of (**a**) H363 and JUJ, (**b**) F379 and 9BY, and (**c**) H437 and 8ZM interactions. The green dashes represent the hydrogen bonding. The schemes of the atom and protein colors are the same as in Figure 5.

Aromatic amino acids, H363, F379, and H437, which evidently affected binding with the individual inhibitors, were also considered (see Figure 3), although these interactions were not observed experimentally [10,11,12,13]. In particular, the binding free energy contribution of JUJ-bound H363 to the complex was the largest, predicted to be −4.23 kJ/mol (see Table S5). This strong interaction was mainly derived from the hydrogen bonding formed by the H363 backbone and the amino pyridine ring of JUJ (Figure 7a). Compared to those with the other inhibitors, F379 provided another distinct binding free energy (−5.18 kJ/mol) upon binding with 9BY. The backbone N atom of F379 was involved in the H-bonded contact with the carboxyl group of 9BY (Figure 7b). In addition, in the hcGAS–8ZM interaction shown in Figure 7c, a hydrogen bond was formed between the side chain of H437 and the tetrazolo-pyrimidine of 8ZM, leading to a larger binding free energy of −5.07 kJ/mol, whereas the binding of H437 to JUJ and ER9 contributed to moderate binding free energies of −2.99 and −2.57 kJ/mol, respectively.

#### 2.5.5. Analysis of the Sandwiched Structure

**Figure 8:**
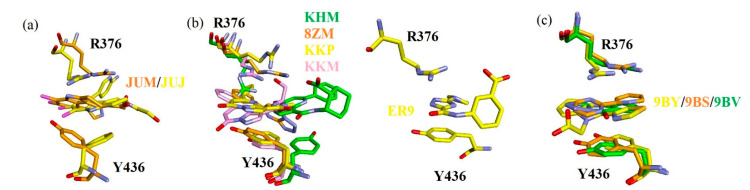
Superposition of sandwiched structures of the inhibitors (**a**) JUM and JUJ, (**b**) KHM, 8ZM, KKP, KKM, and ER9, and (**c**) 9BY, 9BS, and 9BV between the guanidinium group of R376 and the phenyl ring of Y436. Note that the C atoms of the inhibitors and associated proteins are shown in the same color.

Studies have reported that the sandwiched structures formed by the inhibitors with R376 and Y436 play vital roles in cGAS activity [10,11,12,13]. Therefore, we analyzed the binding modes of inhibitors bound to the two residues R376 and Y436 in Figure 8. Generally, the sandwiched structures were predicted by the aromatic rings of the bound inhibitors between the guanidinium group of R376 and the phenyl ring of Y436. For example, Figure 8a shows the superposition of the sandwiched structures in JUJ and JUM between R376 and Y436. The JUJ and JUM structures do not overlap completely, and the side chains of both R376 and Y436 formed π−interactions with the inhibitors in different directions, which was in agreement with the experimental observations [12]. Studies have also reported that the two inhibitors, JUJ and JUM, were inserted in the hydrophobic pocket containing F488 and L490, whereas the binding free energies of F488 and L490 were predicted to be −1.13 kJ/mol and −1.40 kJ/mol, respectively (see Table S5). Figure 8b shows that KHM was located in a deeper pocket than the other inhibitors, resulting in the inability of the ligand to superpose. The diazolo-/tetrazolo-pyrimidine aromatic cores of 8ZM and KKM were sandwiched between R376 and Y436, while the side chains of R376 tended to form hydrogen bonds and cation· · ·π interactions with KHM and KKP (see Figure 5), which was in good agreement with experimental data [11]. Simultaneously, one 1,2,4-triazole core of ER9 was sandwiched between R376 and Y436, consistent with the crystal structure [13]. Compared to bound 9BS and 9BV, 9BY-bound R376 adopted a different alignment to form sandwiched structures in combination with the phenyl group of T436 (Figure 8c), which was consistent with the experimental data [10].

#### 2.5.6. Binding Mechanics

**Figure 9:**
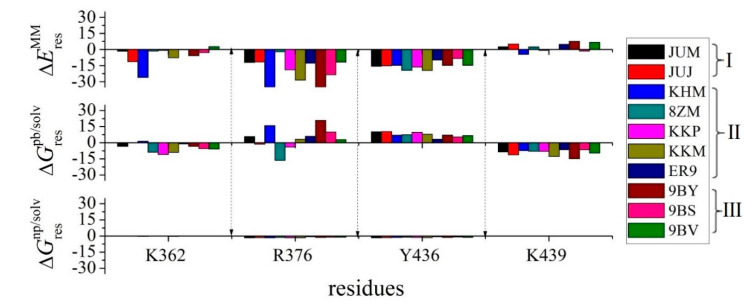
Energy decomposition on the single residues K362, R376, Y436, and K439. Compared to the nonbonded interaction and polar solvation energy, nonpolar solvation energy are almost ignored.

The details regarding the binding mechanics of the three categories of inhibitors with pivotal residues (K362, R376, Y436, and K439) are shown in Figure 9 in terms of single residue binding free energy obtained from energy decomposition analysis. For JUM and JUJ, which have tricyclic pyridoindole cores (category I), the free energies of K362 and R376 binding with JUJ were −7.0 kJ/mol stronger than those of binding with JUM (Table S5), which was due to the strong nonbonded interactions (∆*E*_MM_) and weak polar solvation energy between K362 and JUJ. This also confirmed the C−H · · · π interaction between K362 and JUJ, and the absence of direct contacts with JUM (Figure 4 and Figure S6). In category II, which contained five inhibitors (KHM, KKP, KKM, 8ZM, and ER9), KHM-bound K362 and R376 showed higher binding affinities compared to those bound to 8ZM, KKP, KKM, and ER9 (Table S5). Figure 9 shows that the nonbonded interactions of KHM with K362 and R376 contributed the most to the free energy (−25.90 and −46.42 kJ/mol, respectively). Furthermore, extremely weak nonbonded interaction and polar solvation energy of K362 and K439 with ER9 were observed, which led to weak binding contributions of −1.0 kJ/mol (see Table S5). The binding free energies of the four residues with 8ZM, KKP, and KKM were generally comparable. Nevertheless, the polar solvation energy between R376 and 8ZM contributed attractive energy of −16.29 kJ/mol (Figure 9). For the remaining three inhibitors, 9BY, 9BS, and 9BV, the binding affinity between 9BY and R376 was predicted to be −22.63 kJ/mol (Table S5), which was derived from a strong nonbonded contribution of −42.06 kJ/mol. Compared to 9BS and 9BV, only 9BY formed H-bonded contacts with R376 (Figure 5). Figure 6 shows that one π· · ·π stacking was formed by 9BS upon binding to Y436, while multiple π· · ·π stacking and O−H · · · π interactions were formed by 9BV/9BY upon binding to Y436, leading to stronger nonbonded interactions for 9BV and 9BY at around −15.0 kJ/mol (Figure 9). Despite the absence of direct connections between K439 and all the inhibitors (Figure S6), distinct binding contributions mainly arose from the polar solvation energy with comparable attractive contributions (Figure 9). Although the MM/PBSA technique holds limitations in estimating the binding free energy of the protein-ligand interaction [22], the above results aid in interpreting the experimental observations and offer additional insights from energy decomposition analysis, which is not available in the experimental studies.

## 3. Materials and Computational Methods

### 3.1. Systems

The crystal structures of hcGAS inhibitors were retrieved from the RCSB Protein Data Bank (PDB) from the entries of 6MJW, 6MJU, 6NAO, 6NFO, 5VDU, 6NFG, 5VDV, 5V8O, 5VDW, and 6LRL [27]. The chemical structures of inhibitors bound to hcGAS are shown in Figure 1. The sequences of the inhibitor bound hcGAS proteins were almost identical, except for the mutant residues (K427E/K428E) in 6MJW and 6MJU (see Figure S7). The hcGAS-inhibitor structures were treated as one-to-one forms with an identical residue fragment of 161−522 in this study. Several missing residues of the initial crystal structures were predicted based on the different templates shown in Table S6 using the MODELLER module of the Chimera software [28,29]. Water was retained in the crystal structure to avoid potential interactions between water and the hcGAS-inhibitor systems. Before performing simulations, the protonation states of the charged amino acids were estimated using the H++ web-based tool [30]. In addition to the 4 deprotonated residues H390, C396, C397, and C404, in the zinc-thumb domain of hcGAS, histidines at positions 363 and 390 in 6MJW, 6MJU, 6NFO, and 5VDW were protonated in the N_δ_ atom; the other charged amino acids were treated as default based on H++ analysis.

### 3.2. MD Simulations

The Gaussian 16 program was used to calculate the electrostatic potential (ESP) charges for each inhibitor at the HF/6–31G* level [31]. Restraint ESP charges were derived based on the ESP-fit charge model in AMBER20 [32]. All bonding and van der Waals parameters of the inhibitors were generated from the general amber force field [33]. Each hcGAS-inhibitor complex was placed in the center of a cubic box of 708 to 808 nm^3^ volume and then solvated with 23,640 to 25,860 water molecules using the transferable intermolecular potential with 3 points (TIP3P) water model [34]. Na^+^ and Cl^−^ counterions were added to neutralize the system with salt concentrations consistent with the ones in experimental studies. The AMBER14SB force field was used to calculate the hcGAS-inhibitor energies for the simulations [35]. After preparing the structural parameters, energy minimizations were performed for each simulation system with a maximum of 5000 steps, followed by 2 short 1 ns simulations with position restraint for the heavy atoms and force value of 1000 kJ/(mol∙nm^2^) in the NVT and NPT ensembles at 300 K and 1 bar. Finally, 3 separate 20 ns MD runs were performed for equilibrium with a time step of 2 fs. Temperature coupling was performed using a velocity-rescaling thermostat [36,37]. The Parrinello–Rahman approach was applied under constant pressure control [38,39]. The covalent bonds involving hydrogen were constrained using the SHAKE algorithm [40,41]. The particle mesh Ewald method was used for treating long-range electrostatic interactions [42]. The cut-off distance of van der Waals (vdW) and electrostatic forces were set to 1.2 nm. The snapshots were saved every 100 ps for further structural analysis. These simulations were performed using the GROMACS 2019 package [43].

### 3.3. MM/PBSA Calculations

g_mmpbsa is a fast and reliable tool for estimating the binding free energy based on GROMACS and APBS programs, especially for protein-ligand interactions [23]. Here, the MM/PBSA approach, combined with 3 parallel 20 ns MD trajectories, was used to obtain the binding affinities of hcGAS-inhibitor interactions based on the g_mmpbsa program. Twenty structures for each complex were used to estimate the binding free energy in terms of every 1 ns extraction of the system coordinate. The binding free energy ∆*G*_bind_ of the hcGAS-inhibitor interaction can be evaluated using the following 3 functions:

(1) $$\Delta G_{\mathrm{bind}}=\Delta E_{\mathrm{MM}}+\Delta G_{\mathrm{sol}}-T\Delta S,$$

(2) $$\Delta E_{\mathrm{MM}}=\Delta E_{\mathrm{vdW}}+\Delta E_{\mathrm{ele}}+\Delta E_{\mathrm{bonded}}$$

(3) $$\Delta G_{\mathrm{sol}}=\Delta G_{\mathrm{pb}/\mathrm{solv}}+\Delta G_{\mathrm{np}/\mathrm{solv}}$$

where ∆*E*_MM_ represents the sum of the bonded (∆*E*_bonded_) and nonbonded (∆*E*_vdW_ and ∆*E*_ele_) terms. In the single-trajectory setup, ∆*E*_bonded_ is always assumed to be zero owing to its accuracy and simplicity, which are close to those of the multi-trajectory approach [44]. The solvation free energy ∆*G*_sol_ includes polar solvation energy (∆*G*_pb/solv_) and nonpolar solvation energy (∆*G*_np/solv_), which were estimated using the PB Equation and the solvent-accessible surface area model [45,46]. The default polar and nonpolar parameter settings were identical to those reported previously [23,47]. *T* and ∆*S* are the absolute temperature and entropy, respectively; the entropy calculations were ignored in the current calculation process, as they were time-consuming and improved the experimental data only slightly [18,23,48].

The calculations regarding per-residue binding free energy decompositions were also performed to assess the effect of each residue on the hcGAS-inhibitor interaction. The single residue-free energy was evaluated using Equation (4):

(4) $$\Delta G_{\mathrm{res}}^{\mathrm{bind}}=\Delta E_{\mathrm{res}}^{\mathrm{MM}}+\Delta G_{\mathrm{res}}^{\mathrm{pb}/\mathrm{solv}}+\Delta G_{\mathrm{res}}^{\mathrm{np}/\mathrm{solv}}$$

## 4. Conclusions

MD simulations and MM/PBSA analyses were performed to analyze the binding of various inhibitors (JUM, JUJ, KHM, 8ZM, KKP, KKM, ER9, 9BY, 9BS, and 9BV) with hcGAS. RMSD analysis at approximately 0.25 nm indicated the convergence of all the hcGAS inhibitors. Following this, the binding free energies of hcGAS-inhibitor interactions were estimated using MM/PBSA calculations, which were generally consistent with the experimental affinities, IC_50_ or *K*_d_. We determined the binding free energies at different time scales for every 2 ns, showing correlation coefficients of 0.67 (*r*) and 0.46 (*ρ*). Furthermore, energy decomposition revealed that total nonpolar energy contributed significantly to the total binding energy of each system, where the vdW interaction was stronger than that of the electrostatic interactions. To identify the residue distinctly affecting the inhibitor, we performed per-residue binding energy analysis and investigated the binding modes of the hcGAS-inhibitor complexes. In addition to K362, R376, and Y436 were the major residues that interacted with the inhibitors, which was consistent with the experimental data. New potential roles of the polar residue K439 and aromatic residues H363, F379, and H437 in various interactions, such as hydrogen bonding, salt bridge, π· · ·π stacking, cation· · ·π, O−H· · ·π, and C−H· · ·π interactions, were also revealed. In addition, we analyzed the sandwiched structures of the inhibitor bound to the guanidinium group of R376 and the phenyl ring of Y436; our results suggested that the mechanism of binding mainly involved the impacts from nonbonded interaction and polar solvation energy. Our study indicated that MM/PBSA, in combination with high-throughput virtual screening methods, could be a promising method to screen new inhibitors from the existing database of small molecules. Furthermore, the vital binding modes of hcGAS to inhibitors could be helpful in selecting inhibitors by visual inspection. However, in the current study, the entropy involved in hcGAS-inhibitor interactions was not considered; a potential improvement with the inclusion of entropy should be tested in the future. Moreover, recently, machine-learning algorithms were successfully employed together with MM/PBSA results to achieve a better prediction of the binding free energy of three kinases [49]. A specific machine learning-based predictive model may also be developed in combination with MM/PBSA to improve the estimation of the binding affinity of hcGAS inhibitors.

## Figures and Tables

**Table 1:** Averaged root mean square deviation (RMSD) (nm) evaluations for hcGAS, inhibitor, zinc-thumb domain, and whole structures in 10 hcGAS-inhibitor systems.

Inhibitor Name	hcGAS	Inhibitor	Zinc-Thumb	Whole	PDB ID
JUM	0.243 ± 0.023	0.097 ± 0.044	0.021 ± 0.009	0.243 ± 0.023	6MJU
JUJ	0.250 ± 0.021	0.070 ± 0.018	0.027 ± 0.009	0.250 ± 0.021	6MJW
KHM	0.239 ± 0.028	0.065 ± 0.017	0.051 ± 0.028	0.243 ± 0.027	6NAO
8ZM	0.256 ± 0.019	0.037 ± 0.027	0.035 ± 0.014	0.256 ± 0.019	5V8O
KKP	0.243 ± 0.029	0.046 ± 0.020	0.028 ± 0.010	0.243 ± 0.029	6NFG
KKM	0.241 ± 0.023	0.069 ± 0.021	0.033 ± 0.014	0.241 ± 0.023	6NFO
ER9	0.253 ± 0.020	0.116 ± 0.056	0.041 ± 0.017	0.266 ± 0.032	6LRL
9BY	0.244 ± 0.025	0.089 ± 0.029	0.017 ± 0.012	0.245 ± 0.023	5VDW
9BS	0.274 ± 0.030	0.017 ± 0.046	0.025 ± 0.009	0.275 ± 0.030	5VDU
9BV	0.242 ± 0.024	0.014 ± 0.049	0.033 ± 0.010	0.242 ± 0.024	5VDV

**Table 2:** The averaged binding free energies, ∆*G*_bind_ (kJ/mol), with the standard error of the mean for 10 hcGAS-inhibitor systems obtained from MM/PBSA calculations, along with other components. Here, ∆*G*_pb_ = ∆*E*_ele_ + ∆*G*_pb/solv_ and ∆*G*_np_ = ∆*E*_vdW_ + ∆*G*_np/solv_.

Inhibitor	∆*E*_vdW_	∆*E*_ele_	∆*G*_pb/solv_	∆*G*_np/solv_	∆*G*_pb_	∆*G*_np_	∆*G*_bind_	IC_50_ (μM)	*K*_d_ (μM)
JUM	−150.2 ± 2.2	−40.5 ± 9.6	134.6 ± 9.8	−17.3 ± 0.2	94.1 ± 9.7	−167.5 ± 1.2	−73.4 ± 5.3	0.0275 [12]	−
JUJ	−159.0 ± 3.0	−56.6 ± 8.5	130.2 ± 8.5	−18.4 ± 0.2	73.7 ± 8.5	−177.4 ± 1.6	−103.8 ± 5.5	0.0102 [12]	−
KHM	−155.7 ± 3.5	−144.0 ± 10.3	164.1 ± 8.9	−16.5 ± 0.3	20.1 ± 9.6	−172.2 ± 1.9	−152.2 ± 5.5	2.0/4.9 [11]	0.2 [11]
8ZM	−124.1 ± 2.2	−26.7 ± 4.5	68.6 ± 5.2	−12.0 ± 0.1	41.9 ± 4.8	−136.1 ± 1.1	−94.3 ± 3.4	78 [11]	171 [11]
KKP	−145.6 ± 2.7	−52.9 ± 6.2	92.6 ± 6.4	−14.8 ± 0.2	39.7 ± 6.3	−160.3 ± 1.5	−120.6 ± 5.6	69/125 [11]	78 [11]
KKM	−147.6 ± 4.1	−87.6 ± 10.1	129.6 ± 7.0	−15.5 ± 0.3	42.0 ± 8.5	−163.1 ± 2.2	−121.2 ± 6.5	8.1/17.5 [11]	2.7 [11]
ER9	−134.0 ± 3.2	−81.8 ± 5.7	176.1 ± 7.4	−13.8 ± 0.2	94.3 ± 6.6	−147.9 ± 1.7	−53.8 ± 4.9	13.1 [13]	−
9BY	−132.6 ± 2.8	−117.4 ± 5.5	148.8 ± 6.0	−12.7 ± 0.2	31.4 ± 5.8	−145.3 ± 1.5	−114.1 ± 4.3	−	236 ± 19 [10]
9BS	−92.9 ± 2.7	−35.1 ± 4.1	73.3 ± 5.0	−10.5 ± 0.2	38.2 ± 4.6	−103.4 ± 1.5	−65.3 ± 3.6	−	64 ± 3 [10]
9BV	−110.1 ± 1.9	−14.3 ± 4.5	94.9 ± 4.4	−9.9 ± 0.2	80.6 ± 4.5	−120.0 ± 1.0	−39.2 ± 3.6	−	80 ± 4 [10]

**Table 3:** Comparison of experimental binding energy (∆*G*_exp_) and the estimated binding free energy, ∆*G*_bind_(kJ/mol), at different time scales obtained from MM/PBSA analysis, along with Spearman’s rank correlation coefficient (*ρ*) and Pearson correlation coefficient (*r*).

Inhibitor	∆*G*_exp_	0–4ns	4–8ns	8–12ns	12–16ns	16–20ns
JUM	NA ^a^	−51.8	−73.5	−73.2	−76.7	−84.5
JUJ	NA ^a^	−89.9	−90.1	−117.0	−114.7	−110.4
KHM	−38.5	−155.1	−140.8	−165.9	−160.8	−150.9
8ZM	−21.6	−94.1	−93.6	−96.0	−89.8	−95.3
KKP	−23.6	−132.2	−124.8	−107.5	−120.3	−106.6
KKM	−32.0	−115.4	−124.3	−121.7	−122.4	−120.2
ER9	NA ^a^	−49.4	−52.9	−64.0	−58.7	−49.9
9BY	−20.8	−119.1	−114.4	−116.0	−120.5	−110.8
9BS	−24.1	−62.4	−67.2	−64.9	−57.6	−66.8
9BV	−23.5	−29.9	−35.5	−46.2	−41.0	−45.1
Pearson’s *r*		0.52	0.53	0.70	0.62	0.67
Spearman’s *ρ*		0.32	0.50	0.46	0.46	0.46

^a^ Not available. The experimental binding free energy, ∆*G*_exp,_ was obtained using the Equation ∆*G*_exp_ = −RTln(1/*K*_d_). The values of the inhibitors JUM, JUJ, and ER9 are not available, as *K*_d_ values are not known.

## Data Availability

The data presented in this study are available in the article and supplementary material.

## Supplementary Materials

The following are available online at https://www.mdpi.com/1422-0067/22/3/1164/s1. RMSD analysis for hcGAS and inhibitors in Figures S1 and S2; correlation plot of the predicted binding free energy between multiple-trajectory and single-trajectory analysis in Figure S3; average energy component distribution as a function of time in Figure S4; binding modes between K362 and some inhibitors in Figure S5; 10 inhibitor compounds bound to K439 in Figure S6; multiple sequence alignment of hcGAS in Figure S7; collection of experimental data regarding hcGAS-inhibitor interactions in Table S1; the binding free energy estimated using MM/PBSA followed with energy decomposition components from their separate trajectories in Tables S2 to S4; the binding free energy of the selected hcGAS residues within 0.6 nm of the corresponding inhibitors in Table S5; information regarding missing residue prediction in Table S6.
