# Supplementary material for: Comparative Study of Interactions between Human cGAS and Inhibitors: Insights from Molecular Dynamics and MM/PBSA Studies
Source: Int J Mol Sci. 2021 Jan 25;22(3):1164. doi: 10.3390/ijms22031164 (PMC7865699; doi:10.3390/ijms22031164)
Supplement: Supplementary file 1 [file ijms-22-01164-s001.pdf]

Supplementary Figures

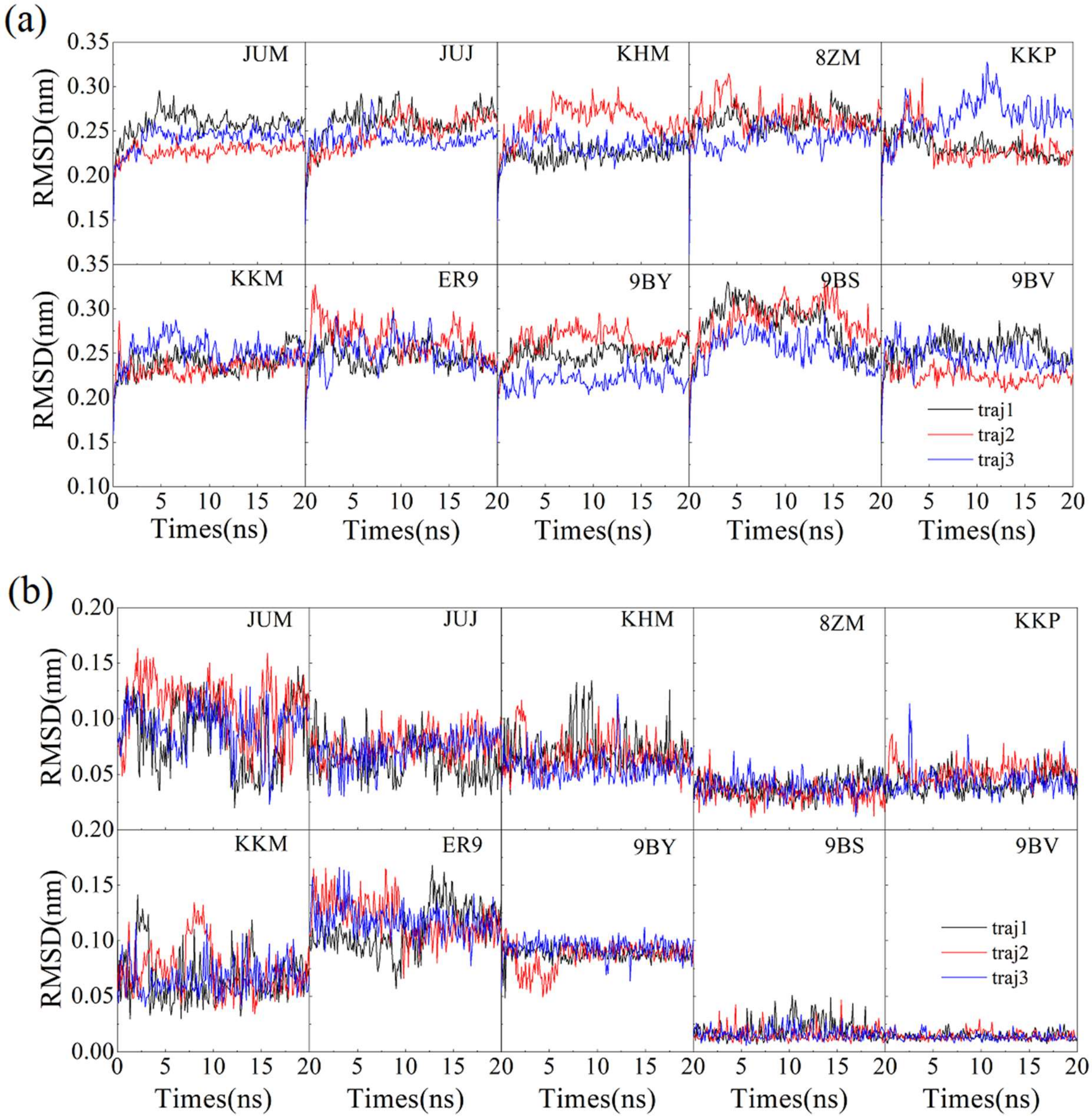

Figure S1. RMSD distributions as a function of time during three parallel MD simulations for (a) isolated hcGAS and (b) bound inhibitor for the hcGAS-inhibitor interactions.

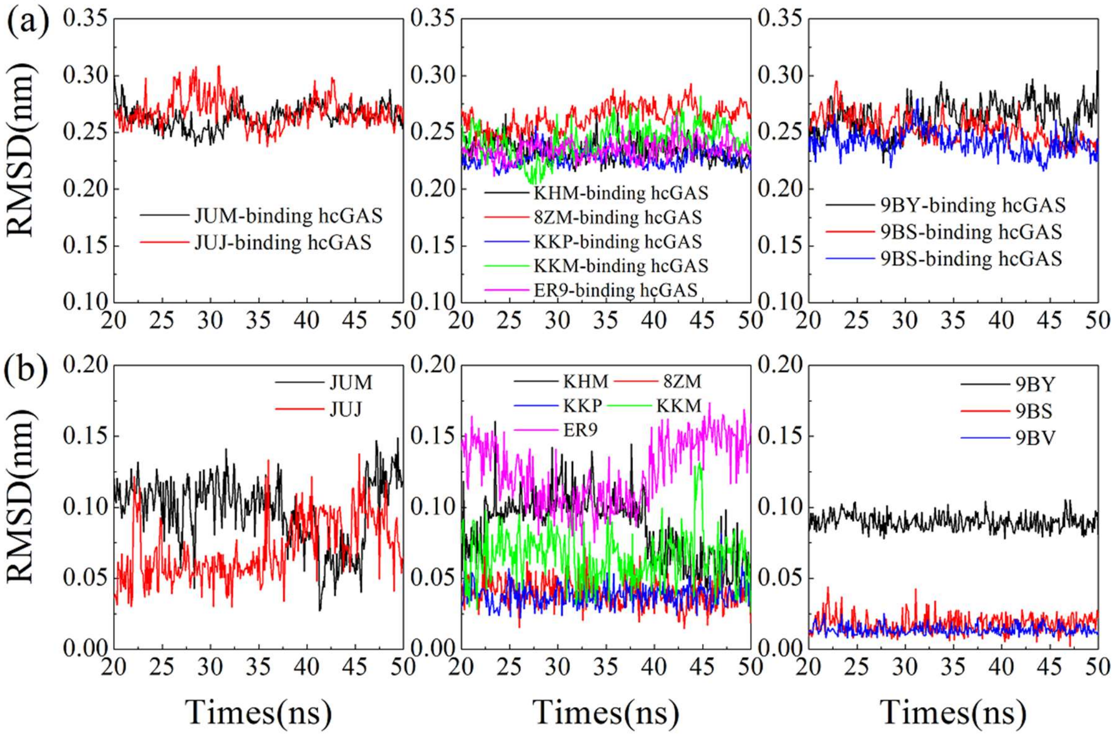

Figure S2. RMSD analyses for the extended 30 ns MD simulations on (a) hcGAS and (b) inhibitors.

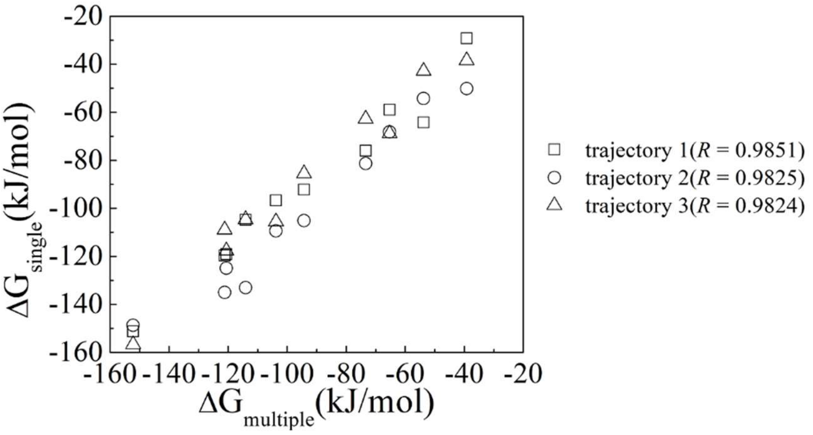

Figure S3. Correlation plot of the predicted binding free energies between multiple-trajectory and single-trajectory analysis. The correlation coefficients *R* are included in the parentheses.

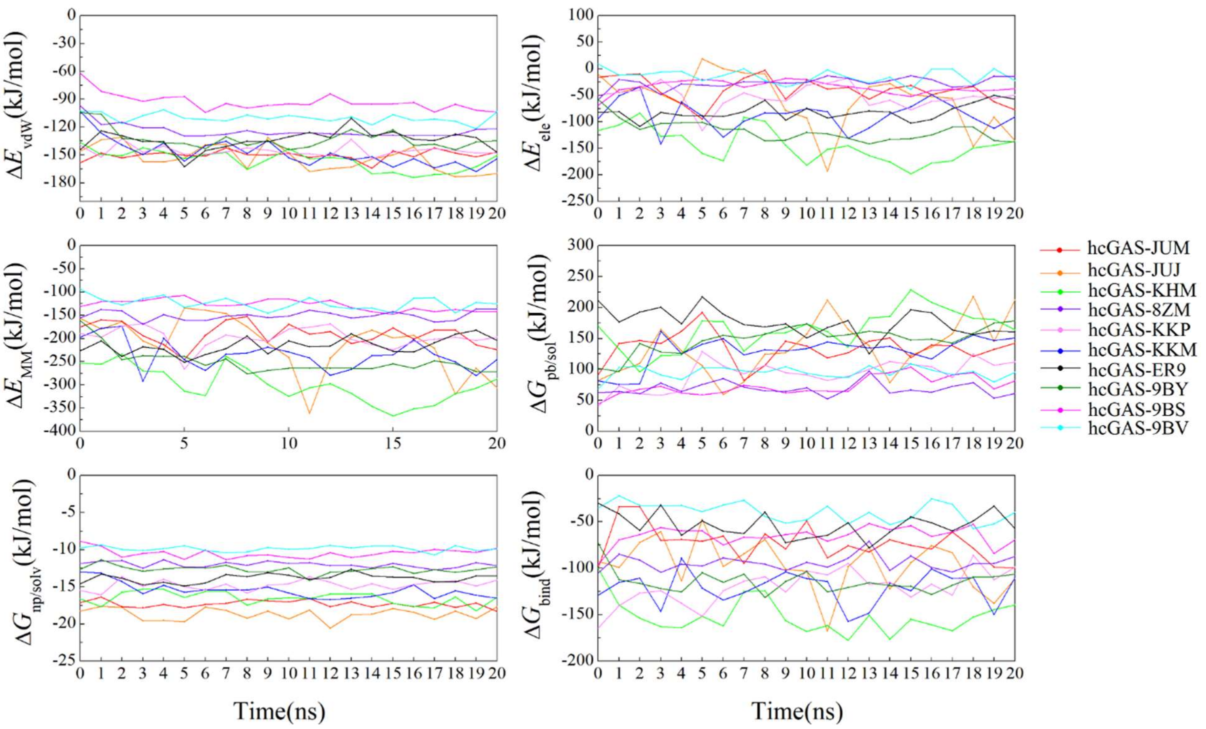

Figure S4. Distribution of all averaged energy components as a function of time, including ΔE_{vdW}, ΔE_{ele}, ΔE_{MM}, $\Delta G_{\mathrm{pb/solv}}$, $\Delta G_{\mathrm{np/solv}}$, and ΔG_{bind} of the ten hcGAS-inhibitor interactions.

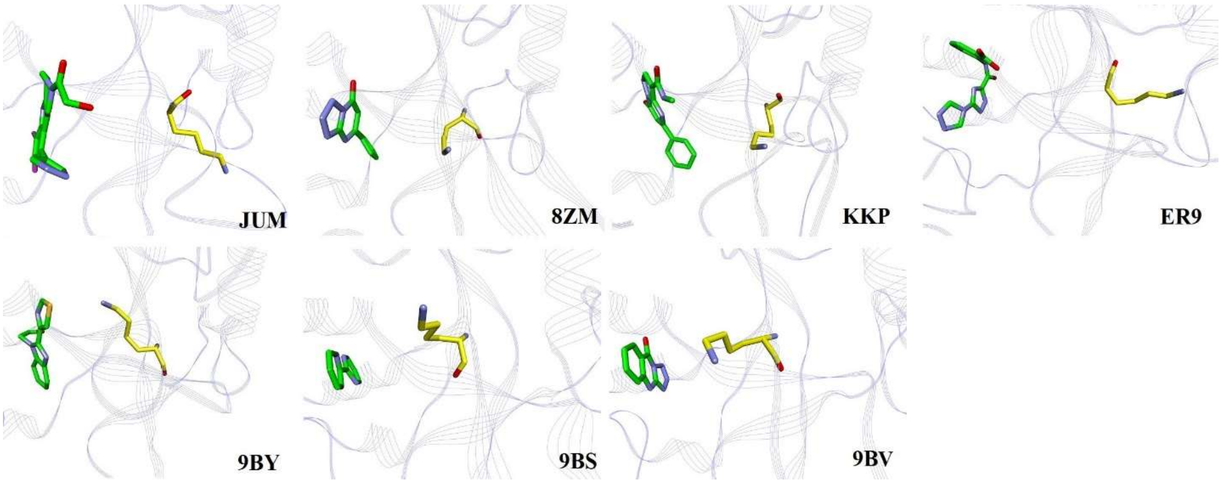

Figure S5. The binding modes between K362 and the inhibitors JUM, 8ZM, KKP, ER9, 9BY, 9BS, and 9BV. The C atoms of inhibitors and residues are shown in green and yellow, respectively. The N, O, and Cl atoms are shown in blue, red, and magenta, respectively. All the hydrogen atoms have been hidden for clarity. The proteins are displayed as purple line ribbon.

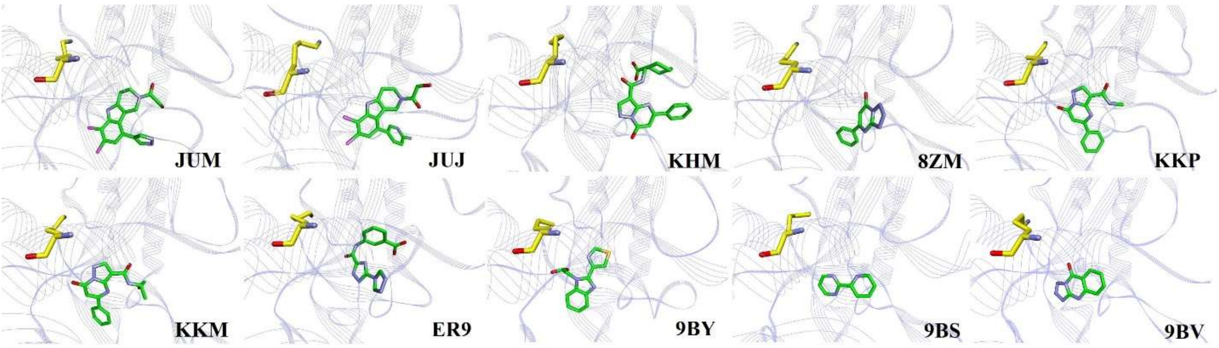

Figure S6. Binding of the ten inhibitors with K439. The C atoms of the inhibitors and K439 are shown in green and yellow, respectively. The atom and protein color schemes are identical to those shown in Figure S3.

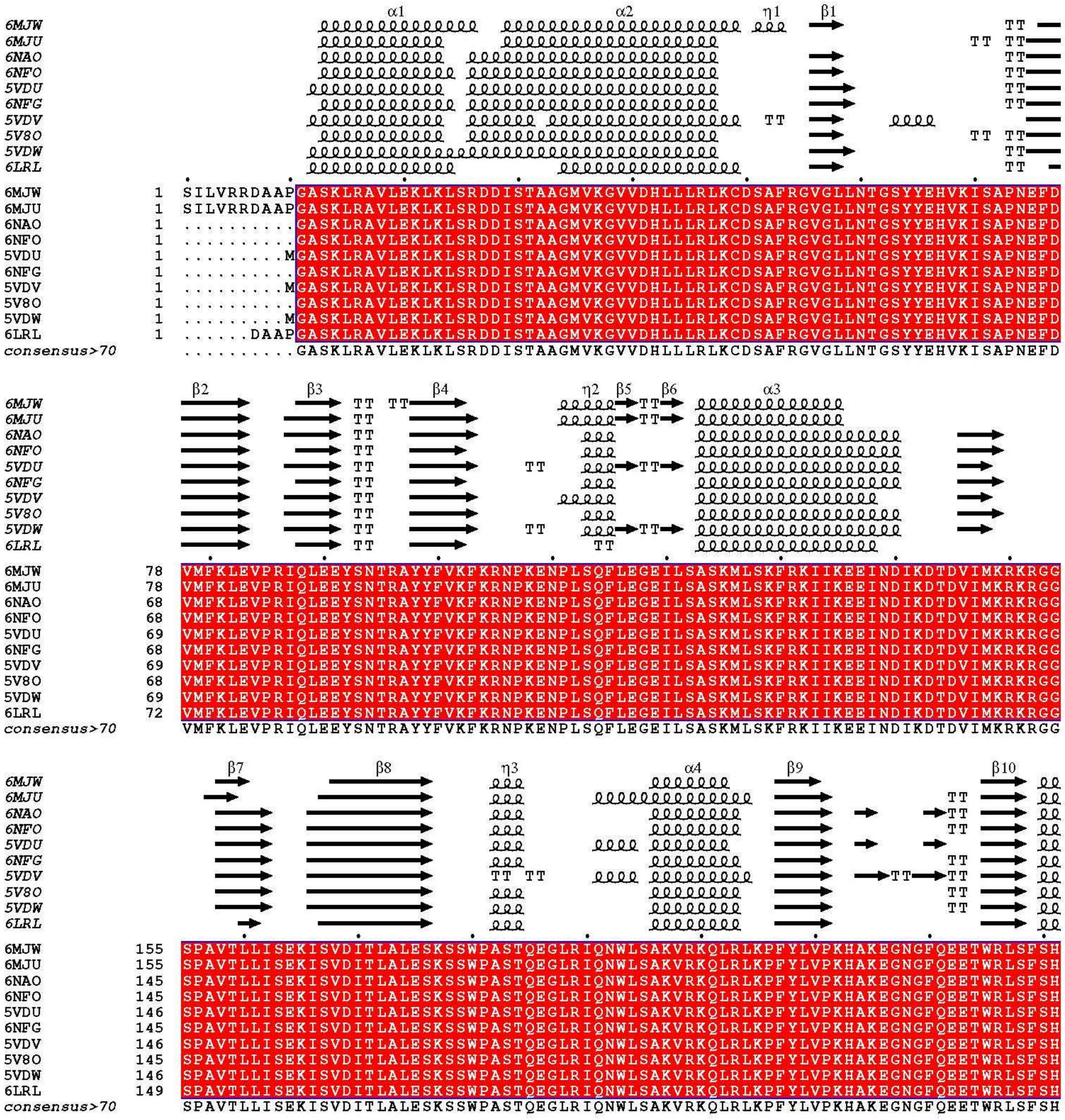

Figure S7 Continued.

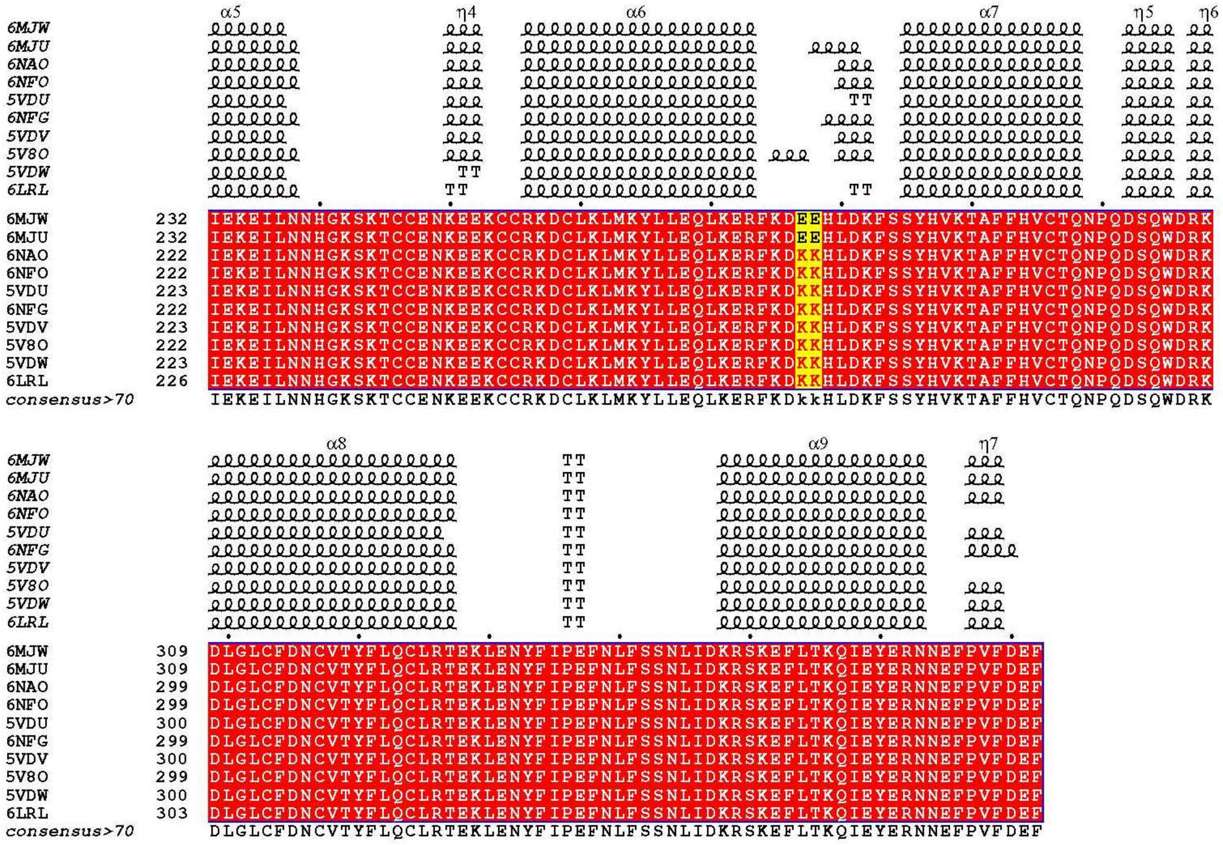

Figure S7. Multiple sequence alignment of the first ten hcGAS proteins obtained using the T–Coffee method. Red boxes represent the identical overlapping regions. The sequences inside the yellow background show the different sequence regions. The secondary structural elements above the aligned sequences were analyzed using ESPript 3.0 webserver. The medium squiggles (α), small squiggles (η), arrows (β), and TT indicate the α-helices, 310-helices, β-strands, and β-turns, respectively.

Supplementary Tables

Table S1. Experimental data regarding binding of hcGAS with the various inhibitors.

No.	PDB ID	Inhibitor name		K_d/μM	IC_{50}/μM	Category
		PDB	reference			
1	6MJU	JUM	G108	–	0.0275	I
2	6MJW	JUJ	G150	–	0.0102	I
3	6NAO	KHM	PF–06928215	0.2	2.0 or 4.9	II
4	5V8O	8ZM	compound 15	171	78	II
5	6NFG	KKP	compound 16	78	69 or 125	II
6	6NFO	KKM	compound 19	2.7	8.1 or 17.5	II
7	6LRL	ER9	compound S2	–	13.1 ± 0.09	II
8	5VDW	9BY	compound F1	236 ± 19	–	III
9	5VDU	9BS	compound F2	64 ± 3	–	III
10	5VDV	9BV	compound F3	80 ± 4	–	III

Table S2. The binding free energy (kJ/mol) estimated using MM/PBSA followed with energy decomposition components from single trajectory 1.

Inhibitor	ΔE_{vdW}	ΔE_{ele}	$\Delta G_{pb/solv}$	$\Delta G_{np/solv}$	ΔG_{pb}	ΔG_{np}	ΔG_{bind}
JUM	-146.4	-47.1	134.7	-17.2	87.6	-163.6	-76.0
JUJ	-153.4	-59.0	134.5	-18.7	75.5	-172.1	-96.7
KHM	-155.1	-106.9	127.1	-16.6	20.2	-171.8	-151.2
8ZM	-123.6	-23.1	66.6	-12.1	43.5	-135.7	-92.2
KKP	-146.3	-43.3	85.8	-14.7	42.5	-161.1	-119.1
KKM	-144.2	-98.3	138.2	-15.0	39.9	-159.2	-119.6
ER9	-153.2	-91.8	195.9	-15.0	104.1	-168.2	-64.2
9BY	-141.6	-149.6	199.6	-13.0	50.1	-154.7	-104.8
9BS	-94.3	-40.0	85.4	-10.2	45.3	-104.4	-58.9
9BV	-110.8	-10.0	101.4	-9.8	91.4	-120.6	-29.2

Table S3. The binding free energy (kJ/mol) estimated using MM/PBSA followed with energy decomposition components from single trajectory 2.

Inhibitor	ΔE_{vdW}	ΔE_{ele}	$\Delta G_{pb/solv}$	$\Delta G_{np/solv}$	ΔG_{pb}	ΔG_{np}	ΔG_{bind}
JUM	-150.1	-58.5	143.4	-16.8	84.9	-167.0	-81.3
JUJ	-167.9	-67.6	144.6	-18.6	77.0	-186.5	-109.4
KHM	-130.4	-161.5	159.2	-15.4	-2.3	-145.8	-148.7
8ZM	-120.4	-18.3	45.3	-11.5	27.0	-132.0	-105.1
KKP	-142.4	-82.0	114.3	-14.9	32.3	-157.3	-124.9
KKM	-149.3	-117.5	148.5	-16.4	30.9	-165.7	-135.0
ER9	-113.6	-93.1	165.0	-12.5	71.8	-126.0	-54.3
9BY	-134.4	-129.4	143.8	-12.6	14.4	-146.9	-133.0
9BS	-98.8	-32.6	73.9	-10.7	41.4	-109.5	-68.2
9BV	-107.0	-22.9	89.3	-9.9	66.4	-116.9	-50.1

Table S4. The binding free energy (kJ/mol) estimated using MM/PBSA followed with energy decomposition components from single trajectory 3.

Inhibitor	ΔE_{vdW}	ΔE_{ele}	$\Delta G_{pb/solv}$	$\Delta G_{np/solv}$	ΔG_{pb}	ΔG_{np}	ΔG_{bind}
JUM	-154.1	-16.1	125.8	-17.9	109.7	-172.0	-62.7
JUJ	-155.9	-43.1	111.6	-17.9	68.5	-173.8	-105.5
KHM	-181.6	-163.7	206.1	-17.5	42.4	-199.1	-156.6
8ZM	-128.3	-38.7	93.9	-12.4	55.3	-140.6	-85.5
KKP	-147.9	-33.2	77.7	-14.7	44.5	-162.5	-117.6
KKM	-149.1	-46.9	102.1	-15.2	55.2	-164.3	-108.9
ER9	-135.3	-60.4	167.3	-14.1	106.9	-149.4	-42.8
9BY	-121.9	-73.3	102.9	-12.4	29.7	-134.3	-104.6
9BS	-85.7	-32.7	60.5	-10.5	27.8	-96.2	-68.8
9BV	-112.6	-10.0	93.9	-9.9	83.8	-122.5	-38.4

Table S5. Binding free energy (kJ/mol) of the selected residues in hcGAS within 0.6 nm of the corresponding inhibitors.

Residue	JUM	JUJ	KHM	8ZM	KKP	KKM	ER9	9BY	9BS	9BV
M229	–0.47	–0.21	–0.45	–0.22	–0.48	–0.43	–0.13	–0.43	–0.37	–0.17
A247	1.17	1.46	–0.30	0.22	3.86	0.94	–0.05	–1.41	–0.42	–0.58
Y248	0.87	1.56	0.74	–0.26	1.26	0.70	–0.47	–0.72	–0.80	–0.24
V360	–0.90	–1.01	–0.55	–0.36	–1.51	–0.86	–0.10	–0.58	–0.84	–0.31
K362	–4.91	–11.61	–24.90	–10.08	–11.84	–16.84	–1.38	–9.14	–8.45	–3.26
H363	–0.48	–4.23	2.43	0.95	0.57	0.87	–0.54	0.57	0.63	0.60
R376	–7.88	–14.38	–32.09	–19.60	–24.33	–26.80	–7.45	–22.63	–14.36	–9.90
L377	1.24	3.43	1.69	7.35	4.13	1.53	1.61	3.54	5.49	2.67
S378	0.83	–0.22	–0.96	–0.57	–1.65	–0.28	0.35	–3.16	–1.13	–0.55
F379	0.17	–0.23	–1.47	–0.59	–0.87	–0.87	0.05	–5.18	–1.22	–1.41
S380	–0.41	–0.34	–0.58	–0.21	–0.68	–1.09	0.17	–1.12	–0.98	–0.42
E383	6.85	6.78	16.30	5.70	9.94	23.45	1.60	13.34	11.63	12.68
K432	–1.12	–3.61	0.26	–4.16	–3.63	–0.67	–3.51	–0.29	–0.44	1.66
F433	–0.37	0.06	–0.96	–0.92	–0.61	–0.78	0.54	–0.76	–0.51	–0.76
S434	–1.61	–2.18	0.41	–1.45	–0.54	–1.22	–1.79	0.74	0.24	1.06
S435	–0.89	–0.65	–1.27	–0.84	–0.70	–1.40	–0.51	–0.15	–0.20	0.31
Y436	–7.00	–6.15	–8.98	–13.03	–8.00	–13.09	–7.08	–9.07	–3.92	–9.09
H437	–0.65	–2.99	–0.99	–5.07	–1.82	–1.66	–2.57	–0.77	–0.61	–0.26
K439	–6.12	–6.13	–11.72	–5.42	–8.86	–12.81	–1.73	–7.25	–7.71	–2.94
N482	3.47	3.12	–1.08	0.97	–1.25	–0.64	0.04	–0.62	–0.14	–1.25
F484	–1.63	–0.87	–1.05	–0.89	–0.97	–1.77	–0.20	–2.24	–1.02	–2.19
I485	–0.97	–0.43	–1.25	–1.60	–2.09	–2.32	–0.03	–2.00	–1.18	–1.50
F488	–2.28	–1.13	–5.30	–2.10	–4.15	–2.31	–0.64	–2.17	–2.51	–1.90
L490	–2.25	–1.40	–2.68	–2.99	–2.13	–0.65	–0.82	–1.66	–2.78	–2.19
L495	–1.84	–2.53	–5.24	–2.09	–1.84	–1.88	–0.48	–1.03	–1.36	–0.33
I496	–0.08	–0.39	–0.33	–0.24	–0.31	–0.27	–0.08	–0.03	–0.10	0.02

Table S6. The missing residues of the ten hcGAS systems and summary of the template and zDOPE score for each target.

target	template	zDOPE	Missing residues
6MJW	4O68, 4KM5	–1.52	161, 255–259, 288–303, 312–315, 365–370, 521–522
6MJU	4O68, 4KM5	–1.51	255–259, 288–303, 311–315, 365–368, 522
6NFO	4KM5	–1.73	254–259, 302–305, 365–567, 521–522
6NFG	4KM5	–1.81	255–260, 303–306, 367–369, 521–522
6NAO	4KM5	–1.79	303–306, 522
5V8O	4O67, 4O69	–1.81	214–216, 303–306, 366–369, 522
5VDV	4O67, 4O69	–1.76	303–306, 521–522
5VDW	4O67, 4O69	–1.84	521–522
5VDU	4O67, 4O69	–1.80	521–522
6LRL	4O68, 4KM5	–1.70	157–160, 174, 198, 210, 216–220, 254–258, 261, 289–292, 299–306, 315, 365–370, 521–522
